# A German Homœopathic Congress

**Published:** 1890-06

**Authors:** Alexander Von Villers

**Affiliations:** Christian Strasse, Dresden, A., Saxony


### A GERMAN HOMŒOPATHIC CONGRESS.

(Schriftleitung der Allgemeinen Homœopathischen Zeitung.)

CHRISTIAN STRASSE, DRESDEN, A., SAXONY, April 9th, 1890.

EDITOR OF THE HOMŒOPATHIC PHYSICIAN:—The "Homœopathische Centralverein Deutschlands," the oldest European homœopathic society, will meet this year in Dresden, on the 9th and 10th of August.

On the 9th we will visit together Meissen, and the house where Hahnemann was born, and on the 10th, after the scientific session, we will dine with our friends at the Belvedere, in Dresden.

Dr. Kafkasen, from Prague, will be chairman.

We would be very happy if some of our American friends, if they are in Europe, would join us, and therefore I ask you to publish a notice of our session in your journal.

As member of the local committee, I beg you to send me word if any one wishes to attend our meetings, and especially if he wish that rooms be secured for him.

With kindest regards,

Most truly yours,

DR. ALEXANDER VILLERS.

